# Long-Term Employment Outcomes among Female Cancer Survivors

**DOI:** 10.3390/ijerph17082751

**Published:** 2020-04-16

**Authors:** Christine C. Ekenga, Eunsun Kwon, BoRin Kim, Sojung Park

**Affiliations:** 1 Brown School, Washington University in St. Louis, St. Louis, MO 63130, USA; spark30@wustl.edu; 2 Department of Social Work, St. Cloud State University, St. Cloud, MN 56301, USA; ekwon@stcloudstate.edu; 3 Department of Social Work, University of New Hampshire, Durham, NH 03824, USA; BoRin.Kim@unh.edu

**Keywords:** cancer, return to work, women, survivorship

## Abstract

Advances in early detection and treatment have led to a growing population of female cancer survivors, many of whom are of working age. We examined the relationship between cancer and long-term (>5 years) employment outcomes in a nationally representative sample of working-age women in the United States. Data from nine waves of the Health and Retirement Study were used to examine employment status and weekly hours worked among cancer survivors (*n* = 483) and women without cancer (*n* = 6605). We used random slope regression models to estimate the impact of cancer and occupation type on employment outcomes. There was no difference in employment status between cancer survivors and women without cancer at baseline; however, during follow-up, cancer survivors were more likely to be employed than women without cancer (odds ratio (OR) = 1.33, 95% confidence interval (CI): 1.11–1.58). Among 6–10-year survivors, professional workers were less likely (OR = 0.40, 95% CI: 0.21–0.74) to be employed than manual workers. Among >10-year survivors, professional workers averaged fewer weekly hours worked (−2.4 h, 95% CI: −4.4–−0.47) than manual workers. The impact of cancer on long-term employment outcomes may differ by occupation type. Identifying the occupation-specific mechanisms associated with the return to work will be critical to developing targeted strategies to promote employment in the growing female cancer survivor population.

## 1. Introduction

In the United States (U.S.), adult cancer survival rates have improved markedly over the past four decades [1]. Driven primarily by improved methods for detection and treatment, survival rates have increased by almost 40% since the late 1970s [1,2]. Currently, approximately 67% of cancer patients survive five or more years after diagnosis [1,2], and survival rates are expected to continue to increase, because of ongoing advances in early diagnosis and treatment options and public health intervention efforts [1,2]. In fact, over the next decade, the number of cancer survivors is projected to increase by 29% and comprise approximately 5% of the U.S. population [3]. 

The late and long-term effects of cancer range from physical to psychosocial and economic [4]. Cancer survivorship has been associated with poor quality of life, fatigue and impaired physical functioning, depression, and cognitive impairment [4,5,6,7,8]. The adverse health effects of cancer can negatively influence the economic well-being of long-term survivors. The economic burden of cancer ranges from the expenses associated with the treatment and management of cancer, to the loss of income and opportunities associated with declines in work productivity [4,9,10,11,12]. However, in comparison to long-term physical and psychosocial outcomes, the economic sequelae associated with cancer and its related treatments are less well investigated. Recent evidence suggests that cancer survivors have higher medical costs, higher debt rates, and higher rates of bankruptcy than those who have never had cancer [13]. Furthermore, the economic burden of cancer may have an impact on survivorship, as material hardship has been associated with adverse physical and mental health outcomes in survivors [14,15,16].

This study focuses on employment outcomes among female cancer survivors. Among U.S. adults, women are more likely to survive cancer than men [17]. As a result of these differences in survival, and in addition to gender differences in employment patterns and occupation, the post-diagnosis employment experiences of women may differ from men. Several previous studies have examined the association between cancer and employment outcomes (e.g., employment status, hours worked per week, schedule changes, earnings, etc.) among women. However, results have been mixed [18,19,20,21,22,23,24,25,26,27,28,29], and only a limited number of long-term (>5 years) studies have investigated the role of occupation type in women’s employment outcomes after cancer. In a Danish population-based study, cancer survivors had a higher likelihood of unemployment during the 20 years of follow-up than persons without a cancer history, and manual work was a significant predictor of unemployment [28]. Another study of Danish breast cancer survivors with an average of 10 years of follow-up reported that manual workers were more likely to experience unemployment than management and office workers [23]. To date, only one U.S. study has reported an association between occupation type and employment outcomes. In a five-year prospective study of cancer survivors from Maryland and Pennsylvania, survivors in physically demanding jobs were more likely to claim disability, but not more likely to be unemployed during follow-up than survivors in less demanding jobs [29].

Given the paucity of long-term (>5 years) studies in the U.S., the objective of the current study was to evaluate the impact of cancer on long-term employment outcomes among middle-aged working (51–64 years) women. We examined predictors of employment status and weekly hours worked, respectively, among U.S. women with and without a history of cancer, with a focus on the predictive effect of occupation type. 

## 2. Materials and Methods 

### 2.1. Study Design

The Health and Retirement Study (HRS) is a longitudinal panel survey of adults aged 51 and older in the United States, that began in 1992 [30]. Conducted by the University of Michigan with support from the National Institute of Aging, the HRS uses a multistage area probability sample to obtain a nationally representative sample of non-institutionalized adults. The HRS was approved by the Institutional Review Board at the University of Michigan, and written informed consent was obtained from all participants. The HRS re-interviews respondents biennially. The data used in this study is de-identified and publicly available through the University of Michigan at http://hrsonline.isr.umich.edu/. 

The HRS has several strengths, including a large sample of female cancer survivors, a prospective study design, and a long follow-up period. The HRS collected detailed information on occupation, which allowed us to evaluate the impact of occupation type on employment outcomes. In addition, our study included a sizeable sample of women without a history of cancer. By including these women, we were able to control for contextual factors, such as economic booms and recessions.

### 2.2. Study Sample

We used nine waves of panel data from the 1998–2014 Health and Retirement Study (HRS). Beginning in 1998, the HRS was combined with the Study of Aging and Health Dynamics and two additional cohorts (Children of the Depression Age (born 1924–1930) and War Babies (born 1942–1947)), which is why we used 1998 as the baseline for our analysis. At the time of our analysis, the HRS provided only an early-release version of 2016 data sets, which did not include income and some health information, resulting in restricting the sample to those observed between 1998 and 2014.

From the respondents who ever responded to surveys during our study period (226,407 observations), we used the following criteria to draw our sample. First, proxy respondents (64,209 observations; 28.3%) and male respondents (65,096 observations; 28.7%) were excluded. Second, the sample excluded adults aged 65 and over (57,563 observations; 25.4%), because of potential differences in employment status and access to Medicare and Social Security. We then excluded adults with cancers diagnosed before 1990 (1251 observations; 0.55%), because of potential differences overtime with regard to cancer screening and treatment protocols [18,31]. Next, given our focus on employment after cancer diagnosis, we excluded individuals’ observations (2340 observations; 1.03%) before their cancer diagnosis. Lastly, we excluded people who never worked during the observation period (9403 observations; 4.15%) and who did not provide work information after diagnosis (41 observations; 0.02%). Listwise deletion for missing information on either the dependent or the explanatory variables led to further reductions (149 observations; 0.06%), resulting in a total unweighted sample of 7088 individuals (26,355 observations; 11.64%): 483 cancer survivors (1709 observations) and 6605 individuals without a cancer history (24,646 observations).

The retention of healthier respondents overtime in longitudinal analyses can lead to biased results that favor more robust cancer survivors. To control for bias in follow-up waves, introduced by missing data resulting from death, we created a mortality variable. Of the 101 individuals who died during the study period, 22 individuals were cancer survivors. Including mortality information as a separate control variable during sensitivity tests introduced little change in effect sizes for the other control variables, so this variable was not included in the final models. 

### 2.3. Measures

#### 2.3.1. Employment Outcomes

There were two dependent variables in this study: employment status and hours of weekly work. Employment status was ascertained by asking respondents “Are you doing any work for pay at the present time?” Respondents who responded affirmatively to this question, including full-time (≥35 h per typical work week) and part-time (<35 h per typical work week) workers, were classified as employed. Hours of weekly work was defined as the average hours worked per week.

#### 2.3.2. Cancer Survivorship Status

Respondents were asked, “Has a doctor ever told you that you have cancer or a malignant tumor of any kind except skin cancer?” Respondents who responded affirmatively to this question were categorized as cancer survivors and the rest were categorized as women without a cancer history. Cancer survivors were asked the year they were diagnosed with their most recent cancer. We used this information to determine time since cancer diagnosis, categorized as ≤2 years, 3–5 years, 6–10 years, or >10 years.

#### 2.3.3. Occupation Type

The occupational level was determined on the basis of the current occupation (or the most recent, if not in paid employment) of the respondent. Occupations were classified according to the often-used Erikson-Goldthorpe class scheme [32] into two categories: professional (professionals, managers, other white-collar employees) and manual (farmers, skilled blue-collar employees, and unskilled/low-wage service) occupations.

#### 2.3.4. Covariates

The following covariates were included as potential confounders based on previous literature [18,33,34]: age, race/ethnicity, educational attainment, marital status, minor-aged children in the household, family income as a percent of federal poverty line, and type of health insurance. Treatment-induced symptoms could be associated with employment after cancer [35,36]. Therefore, we included the aforementioned time since diagnosis variable and measures of ongoing cancer treatment at baseline (yes/no), self-rated health (ranging from 1 (excellent) to 5 (poor)), fatigue (yes/no), and depressive symptoms (yes/no) [33,34,37]. The study period included the Great Recession that spanned from 2007 to 2009 and impacted U.S. employment [38]. This time period was coded as 1 (other periods coded as 0) and included in the analyses.

### 2.4. Statistical Analysis

Univariate analyses were used to compare the baseline characteristics of cancer survivors and women without a history of cancer. Overall differences in percentages and standard errors were examined using χ2 tests, and overall differences in means were tested using Wald F-tests. 

We used two sets of analyses to evaluate employment outcomes after cancer: The first set of analyses examined the impact of cancer on employment status and the second set of analyses examined the impact of cancer on the number of work hours for women who were working full-time (≥35 h per week) at baseline (sample of 4627 women with 17,291 observations; 330 cancer survivors and 4297 women without a cancer history). We used multilevel growth modeling to examine how cancer and occupation type affect changes in employment status and hours worked per week overtime. Multilevel growth models are specifically designed for the analysis of trajectories in repeated measures of longitudinal or panel data [39]. They estimate individual trajectories based on person-specific initial values of outcomes (intercepts) and rates of change (slope) that describe intra-individual change patterns in outcome as a function of time. In the current study, the time indicator was determined by the respondents’ wave participation. It was represented as a continuous variable and centered on the grand mean, ranging from −1.84 to 5.16; the intercept represented outcome level at mean centered time of follow-up.

We conducted preliminary analyses to determine the specification of fixed and random effects for change in outcomes overtime (results not shown). For both outcomes, we estimated total constant correlation across occasions and assessed the relative magnitude of each source of variation via an intra-class correlation (ICC). For employment status, the results showed that 42% of differences were due to constant mean differences between persons, while 58% were due to remaining variation surrounding person means. For hours worked per week, 38% of differences were due to the variability between persons. Comparisons of model fit between models of increasing complexity indicated that a random linear model provided the best fit for describing time-related outcome change. 

The repeated observations equation that captured changes in employment status and hours worked associated with time is defined as:

Yit = αi + βiTIMEit + εit(1)

Yit represents the employment status and hours worked for respondent i at time t; αi represents the mean odds of being employed and mean number of hours worked at centered time 1.86 for a respondent i; βi is the mean linear component of the trajectory, indicating the linear rate of change in odds of being employed and in hours worked for respondent i with each additional time; and εit is an error term which is the deviation of each respondent i at time t from their average odds of being employed and level of hours worked. Our data involve equal time intervals between observations (2 years), which could be easily incorporated into the random coefficient growth curve model involving random effects (time) to examine intra-individual differences in initial status and rates of change. The estimation of the fixed and random effects for a specific individual can be expressed as:

αi = α0 + u1i, βi = β0 + u2i(2)

Here, αi can be interpreted as hours worked at time t=0 for a respondent who has an average trajectory (u1i = 0); βi is the expected amount of change in employment status and hours worked per wave (two years), for a respondent who has an average trajectory (u2i = 0). The error terms, u1i and u2i, reflect the amount of variation that exists among individuals in relation to their growth parameters. Combining the Equations (1) and (2), the mixed model of fixed and random effects can be expressed as (3). Here, the former parenthesis is interpreted as the fixed effects (level-1) that estimate repeated observations capturing changes in employment status and hours worked associated with time. The latter parenthesis is the random effects (level-2) that capture intra-individual differences in the initial level and rates of change.

Yit = (α0 + β0TIMEit) + (u1i + u2i TIMEit + εit)(3)

We found evidence of interaction only for occupation type in preliminary analyses. Variables were entered in the following order: Model 1 examined the effect of cancer, Model 2 tested the effect of time since diagnosis, Model 3 added interaction terms between cancer and occupation type, and Model 4 tested for interactions between time since diagnosis and occupation type. 

Finally, to examine employment outcomes among cancer survivors, we carried out separate analyses among cancer survivors only. Coefficients for time were not allowed to vary at the individual level (a random slope model); that is, there was no evidence of variation in individual-level changes. Therefore, we used random effects logistic regression models (xtlogit in STATA, StataCorp LP, College Station, TX, USA) for employment status and random effects regression models (xtreg in STATA) for hours worked per week. These models accounted for the correlation of repeated observations from each individual resulting from the longitudinal design, and included an individual-specific random intercept, allowing us an individual-specific interpretation. All analyses were conducted using the STATA statistical software package (version SE 16.0, StataCorp LP, College Station, TX, USA).

## 3. Results

### 3.1. Characteristics of the Study Sample

Table 1 presents baseline characteristics of the sample and their bivariate associations with cancer survivorship status. Of 7088 middle-aged women, approximately 7 percent were diagnosed with cancer (*n* = 483). The average time since diagnosis was 8.8 years. Cancer survivors were significantly different from women without a cancer history with regard to their race/ethnicity, education, marital status, occupation, household income and health status. Cancer survivors reported more fatigue and depression than women without a cancer history. At baseline, in unadjusted analyses, cancer survivors had a higher percentage of employment and a higher average number of hours worked per week than women without a history of cancer.

### 3.2. Cancer Status and Hours Worked

Table 2 presents estimates from random slope coefficient model of hours worked per week among women who were working full time (≥35 h per week) at baseline. The initial level of hours worked did not vary significantly between cancer survivors and women without cancer, although professional job holders tended to work more hours at baseline and overtime than women with unskilled manual jobs (Model 1). Women who were ≤2 year cancer survivors worked more hours at baseline than women without cancer (Model 2). Women who were >10 year cancer survivors worked more hours than women without cancer at baseline (+4.2, 95% CI = 1.04–7.48) and overtime (+2.3, 95% CI = 0.54–4.09); however, professional women tended to work less hours at baseline (−4.2, 95% CI = −7.60–−0.83) and overtime (−2.4, 95% CI = −4.38–−0.47) than women who had unskilled manual jobs (Model 4). Health status did not significantly impact effect sizes.

### 3.3. Employment Status and Hours Worked among Cancer Survivors

In the supplementary analyses, we examine employment outcomes among only cancer survivors. 

Compared with ≤2 year survivors, 3–5 year survivors (OR = 0.29, 95% CI = 0.10–0.79), 6–10 year survivors (OR = 0.37, 95% CI = 0.14–0.97), and >10 year survivors (OR = 0.24, 95% CI = 0.09–0.66) were less likely to be employed (Model 1 in Table S1). However, these associations were not significant after the addition of the time since diagnosis*occupation type interaction term (Model 2 in Table S1).

With regard to hours worked per week, 3–5 year survivors (−6.0 h, 95% CI =−11.17–−0.76), 6–10 year survivors (−5.6 h, 95% CI = −10.46–−0.70) and >10 year survivors (−6.2 h, 95% CI = −11.09–−1.24) tended to work less hours at baseline than ≤2 year survivors (Table S2). However, after the addition of the time since diagnosis*occupation type interaction term, only 3–5 year survivors (−9.4 h) and 6–10 year survivors (−9.5 h) reported significantly less hours of work than ≤2 year survivors (Model 2 in Table S2).

## 4. Discussion

In this longitudinal study of employment participation among female cancer survivors and women without a history of cancer, we found that, although there were no significant differences between the two groups at baseline, over time, cancer survivors were 33 percent more likely to be employed for pay than women without a cancer history. Among women who were employed full-time at baseline, there were no significant differences in hours worked between cancer survivors and women without a cancer history. However, we observed that the length of time since cancer diagnosis, in conjunction with occupation type, was associated with hours worked; professional women with more than 10 years of cancer survivorship worked, on average, 2.4 h less per week than 10-year cancer survivors in unskilled manual jobs.

We found that cancer survivors were more likely to be employed for pay during the study follow-up period. These findings differ from those of several longitudinal studies, which found that either cancer survivors were less likely to be employed than non-survivors, or that there were no significant differences in employment outcomes between the two groups [18,19,20,21,22,23,28,29]. Additionally, unlike previous studies [26,40], we found that indicators of health status such as fatigue and depression were not associated with employment outcomes. Those prior studies focused on relatively short-term (<5 years) survivor periods, and one possible explanation for our findings is that our sample of cancer survivors were generally long-term survivors with an average of nine years of survivorship at baseline. As noted by Bradley and colleagues in a prior analysis of the HRS [41], there is a potential for selection bias, as long-term cancer survivors are often high socioeconomic status women, who are more likely to be diagnosed with cancer at earlier stages. These long-term survivors may also have been women who have strong affiliations to the labor market, or they may be working to maintain health insurance. As the HRS did not collect data on intentions or reasons to stay in the workforce, job attachment, or the ability to return to work, we were unable to evaluate the role of these factors in employment participation. In addition to evaluating individual intent, future longitudinal studies should evaluate contextual factors, such as job accommodations, job benefits, and employer/co-worker support. Examining the influence of both individual and contextual work factors will be key to advancing our understanding of the impact of cancer on long-term employment outcomes.

To our knowledge, our study is the first to examine the interaction between occupation type and the length of cancer survivorship. We observed that professional cancer survivors with the longest length of survivorship (>10 years) work fewer hours per week than survivors in unskilled manual jobs. Cancer and its associated treatments have been associated with cognitive limitations years after diagnosis [4,8]. Although cancer survivors with professional jobs were more likely to maintain employment than women in unskilled manual jobs, cancer-induced impairment may have made it difficult for these women to meet the cognitive demands of their job. Instead of leaving the workforce, these women could have decided to reduce their work hours to manage their workload and health. Additionally, cancer survivors with professional jobs may have had more flexibility to reduce their work hours than cancer survivors in manual positions.

The limitations of our study should be noted. First, cancer history was ascertained by self-report and not independently validated through medical records. Additionally, information about the site and stage of cancer was not available in the HRS. Cancer is a heterogeneous group of diseases, with prognoses that vary according to tumor characteristics and treatment, and certain cancers may have a stronger influence on employment outcomes than others. In previous studies of employment outcomes by cancer site, women diagnosed with breast and hematological cancers have had better outcomes than women with gynecological and respiratory cancers [21,42,43]. However, it should be noted that the majority of the studies of women’s employment after this have focused exclusively on breast cancer survivors. Although 40% of female cancer survivors are breast cancer survivors, our study’s inclusion of all cancer sites provided a broader understanding of post-cancer employment outcomes among the general female cancer survivor population.

## 5. Conclusions

The results of this study suggest that, although cancer may not have a negative impact on overall employment, the long-term effects of cancer may include reduced work hours for professional women. Our findings highlight the complex relationships between cancer, occupation type, and employment outcomes. For some occupations, instead of a barrier to employment, cancer may represent a barrier to work productivity. As the number of cancer survivors continues to grow, the costs associated with lost productive work time has the potential to be substantial. Further work is necessary to confirm these findings and elucidate pathways to productive employment for cancer survivors.

## Figures and Tables

**Table 1:** Baseline Characteristics of the Study Sample, Health and Retirement Study, 1998–2014 (*n* = 7088).

**Variable**	**Women without Cancer**		**Cancer Survivors**	**t/ꭓ^2 a^**
	**N = 6605**		**N = 483**	
	Mean (SD)/Median (SE)			
Age	54.8 (3.5)		55.6 (3.8)	4.74(1) *
Hours worked per week	33.3 (16.5)		34.6 (17.9)	5.7(1) *
Adjusted Income ($)	56,370 (76,739)		58,493 (52,782)	102.06(1) ***
No. Chronic Diseases	1.02 (1.02)		2.17 (1.17)	17.82(1) ***
Self-rated health	2.53 (1.03)		2.89 (1.07)	1.62(1)
Years since cancer diagnosis			8.8 (5.6)	
	*N* (%) ^b^			
Employment Status				
Employed	6025 (91.2)		442 (91.5)	0.04
Full-time	4297 (69.0)		330 (69.0)	1.96
Part-time	1632 (25.1)		107 (22.4)	
Unemployed	580 (8.9)		41 (8.6)	
Occupation				9.8(1) **
Professional	3590 (54.4)		298 (61.7)	
Non-professional	3015 (45.7)		185 (38.3)	
Race/Ethnicity				10.3(3) *
Non-Hispanic White	4075 (61.7)		333 (68.9)	
Non-Hispanic Black	1432 (21.7)		88 (18.2)	
Hispanic	876 (13.3)		49 (10.1)	
Other	222 (3.4)		13 (2.7)	
Marital Status(married)	4523 (68.5)		310 (64.2)	3.82(1) *
Children in Household(yes)	2793 (42.3)		183 (37.9)	3.57(1)
Education				20.9(4) ***
General Educational Diploma (GED) or less	1218(18.4)		62(12.9)	
High School graduate	1961 (29.7)		153 (31.7)	
Some college	1855 (28.1)		122 (25.3)	
College and above	1571 (23.8)		146 (30.2)	
Household Income as % Federal Poverty Level (FPL)			14.0(3) **	
≤138%	1139 (17.2)		56 (11.6)	
138–250%	1203 (18.2)		94 (19.3)	
250–400%	1325 (20.1)		87 (18.0)	
≥400% Observed during Great Recession	2938 (44.5) 1756 (26.33)		246 (50.9) 125 (25.67)	0.57(1)
Insurance Status				
Public insurance	3050 (46.8)		295 (61.1)	40.5(2) ***
Private insurance	2873 (43.5)		156 (32.3)	
Uninsured	682 (10.3)		32 (6.6)	
Fatigue(yes)	800 (12.1)		102 (21.1)	32.9(1) ***
Depression(yes)	977 (14.8)		85 (17.6)	2.8(1) *
Years since cancer diagnosis				-
≤2 years			62 (12.8)	
3–5 years			101 (20.9)	
6–10 years			149 (30.9)	
>10 years			171 (35.4)	
Currently undergoing cancer treatment(yes)			110 (22.8)	-
**^a^**	**Model 1**	**Model 2**	**Model 3**	**Model 4**
	**Cancer**	**Years since Diagnosis**	**Cancer × Occupation**	**Years since Diagnosis × Occupation**
	OR (S.E.) ^b^	OR (S.E.) ^b^	OR (S.E.) ^b^	OR (S.E.) ^b^
Fixed Effects				
Intercept (baseline level) ^c^	26.92 (4.58) ***	26.97 (4.59) ***	26.61 (4.54) ***	26.60 (4.54) ***
Cancer status (ref: no cancer)				
Has cancer	1.27 (0.19)		1.51 (0.34)	
Years since diagnosis (ref: no cancer)				
≤2 years		2.83 (1.47) *		1.43 (1.11)
3–5 years		1.03 (0.34)		1.49 (0.74)
6–10 years		1.61 (0.41)		2.33 (.95) *
>10 years		0.94 (0.22)		1.13 (0.39)
Occupational status (ref: unskilled/manual)				
Professional occupations	2.57 (0.20) ***	2.56 (0.20) ***	2.61 (0.21) ***	2.62 (0.21) ***
Interaction Terms (cancer × occupation)				
Cancer × professional			0.79 (0.22)	
Interaction Terms (years since diagnosis × occupation)				
≤2 years × professional				2.55 (2.69)
3–5 years × professional				0.50 (0.30)
6–10 years × professional				0.68 (0.33)
>10 years × professional				0.78 (0.32)
Linear slope (ctime)^d^	0.66 (0.02) ***	0.66 (0.02) ***	0.65 (0.02) ***	0.65 (0.02) ***
Cancer status (ref: no cancer)				
Has cancer	1.33 (0.12) **		1.59 (0.22) **	
Years since diagnosis (ref: no cancer)				
≤2 years		3.30 (1.16) **		1.91 (1.03)
3-5 years		1.07 (0.23)		0.87 (0.28)
6-10 years		1.34 (0.20)		2.35 (0.59) **
>10 years		1.18 (0.16)		1.45 (0.29)
Occupational status (ref: unskilled/manual)				
Professional occupations	0.77 (0.03) ***	0.77 (0.03) ***	0.79 (0.03) ***	0.79 (0.03) ***
Interaction Terms (cancer × occupation)				
Cancer × professional			0.74 (0.12)	
Interaction Terms (years since diagnosis × occupation)				
≤ 2 years × professional				2.20 (1.58)
3–5 years × professional				1.48 (0.58)
6–10 years × professional				0.40 (0.12) **
>10 years × professional				0.72 (0.18)
Random Effects				
Variance(ctime)	1.00 (0.08)	0.99 (0.08)	1.00 (0.08)	0.99 (0.08)
Variance(Intercept)	4.53 (0.26)	4.50 (0.26)	4.53 (0.26)	4.51 (0.26)
Covariance (ctime, intercept)	1.35 (0.11)	1.33 (0.11)	1.35 (0.11)	1.33 (0.11)
Log likelihood chi^2^(df)	779.53 (24) ***	789.37 (30) ***	782.62 (26) ***	798.20 (38) ***

^a^ Note: N = 7088 individuals; Observations = 26,355 observations (483 cancer survivors (1709 observations) and 6605 individuals with no cancer history (24,646 observations)). ^b^ Odds Ratios (Standard Error) are based on random slope logistic regression models adjusted for age, race/ethnicity, education, marital status, economic recession experience, children in the household, family income, type of health insurance, self-rated health, fatigue, depressive symptoms, and current cancer treatment; * *p* < 0.05. ** *p* < 0.01. *** *p* < 0.001; ^c^ Intercepts indicate person-specific baseline level (initial values) of employment status. ^d^ Linear slopes describe intra-individual patterns of change in employment status as a function of centered time (ctime).

**Table 2:** Cancer and Weekly Hours Worked among Full-time Employed Women ^a^.

Variable	Model 1	Model 2	Model 3	Model 4
	Cancer ^b^	Years since Diagnosis ^b^	Cancer × Occupation ^b^	Years since Diagnosis × Occupation ^b^
Fixed Effects				
Intercept (baseline level) ^c^	36.10 (0.69) ***	36.12 (0.69) ***	36.02 (0.69) ***	36.02 (0.69) ***
Cancer status (ref: no cancer)				
Has cancer	1.26 (0.69)		2.44 (1.12) *	
Years since diagnosis (ref: no cancer)				
≤2 years		4.84 (2.09) *		5.11 (3.47)
3–5 years		0.80 (1.53)		0.48 (2.57)
6–10 years		0.48 (1.18)		−0.68 (2.16)
>10 years		1.12 (1.06)		4.26 (1.64) **
Occupational status (ref: unskilled/manual)				
Professional occupations	1.72 (0.34) ***	1.72 (0.34) ***	1.85 (0.35) ***	1.84 (0.35) ***
Interaction Terms (cancer × occupation)				
Cancer × professional			−1.58 (1.22)	
Interaction Terms (years since diagnosis × occupation)				
≤2 years × professional				−0.52 (4.26)
3–5 years × professional				0.22 (2.99)
6–10 years × professional				1.44 (2.37)
>10 years × professional				−4.22 (1.72) *
Linear slope (ctime) ^d^	−5.37 (0.16) ***	−5.370 (0.16) ***	−5.43 (0.16) ***	−5.43 (0.16) ***
Cancer status (ref: no cancer)				
Has cancer	0.28 (0.35)		1.22 (0.63)	
Years since diagnosis (ref: no cancer)				
≤2 years		1.99 (1.39)		1.98 (2.49)
3–5 years		−0.49 (0.91)		−1.44 (1.47)
6–10 years		−0.32 (0.64)		0.26 (1.22)
>10 years		0.41 (0.51)		2.31 (0.90) *
Occupational status (ref: unskilled/manual)				
Professional occupations	1.20 (0.18) ***	1.19 (0.18) ***	1.28 (0.19) ***	1.28 (0.19) ***
Interaction Terms (cancer × occupation)				
Cancer × professional			−1.26 (0.72)	
Interaction Terms (years since diagnosis × occupation)				
≤2 years × professional				0.01 (3.01)
3–5 years × professional				1.46 (1.74)
6–10 years × professional				−0.87 (1.39)
>10 years × professional				−2.42 (0.99) *
Random Effects				
Variance (ctime)	13.09 (0.72)	13.04 (0.72)	13.09 (0.72)	13.02 (0.72)
Variance (Intercept)	98.00 (3.46)	97.83 (3.46)	98.00 (3.46)	97.84 (3.46)
Covariance (ctime, intercept)	35.82 (1.43)	35.73 (1.43)	35.82 (1.43)	35.70 (1.43)
Variance (residual)	147.37 (1.84)	147.36 (1.84)	147.34 (1.84)	147.25 (1.84)
Log likelihood chi^2^(df)	2911.44 (24) ***	2921.92 (30) ***	2689.38 (26) ***	2935.47 (38) ***

^a^ N = 4627 (17,291 observations; 330 cancer survivors and 4297 individuals with no cancer history). ^b^ Coefficients are based on random slope coefficient models adjusted for the covariates; * *p* < 0.05. ** *p* < 0.01. *** *p* < 0.001; ^c^ Intercepts indicate person-specific baseline level of weekly hours worked. ^d^ Linear slopes describe intra-individual patterns of change in weekly hours worked as a function of centered time (ctime).

## Supplementary Materials

The following are available online at https://www.mdpi.com/1660-4601/17/8/2751/s1, Table S1: Effect of Length of Cancer Survivorship and Occupation Type on Odds of Employment among Cancer Survivors, Table S2: Effect of Length of Cancer Survivorship and Occupation Type on Weekly Hours Worked among Cancer Survivors.
