# Erratum: Ekenga, C.; et al. Long-Term Employment Outcomes among Female Cancer Survivors. *Int. J. Environ. Res. Public Health* 2020, *17*, 2751

**DOI:** 10.3390/ijerph17249169

**Published:** 2020-12-08

**Authors:** Christine C. Ekenga, Eunsun Kwon, BoRin Kim, Sojung Park

**Affiliations:** 1 Brown School, Washington University in St. Louis, St. Louis, MO 63130, USA; spark30@wustl.edu; 2 Department of Social Work, St. Cloud State University, St. Cloud, MN 56301, USA; ekwon@stcloudstate.edu; 3 Department of Social Work, University of New Hampshire, Durham, NH 03824, USA; BoRin.Kim@unh.edu

Due to an error during production, Section 3.2 in the results section was inadvertently omitted in the published paper [1]. The corrected section is provided below:

*3.2. Cancer and Employment Status*

Table 2 presents estimates from random slope logistic regression models for employment status. In all models, women were less likely to be employed overtime. Significant intra-individual differences in employment status changes were found, with some women experiencing an improvement in employment status and others a decline. Model 1 contains the estimates for the effect of cancer on the initial level and rate of change in employment status, after adjusting for sociodemographic and health-related factors. At baseline, there was no significant difference in employment status between cancer survivors and the comparison group. Over time, however, compared to women without cancer, cancer survivors had a 1.33 times greater likelihood of working (95% Confidence Interval (CI) = 1.11–1.58).

In Model 2, we estimated the effects of time since cancer diagnosis on employment status. Compared with women without cancer, ≤2 year cancer survivors had a 2.8 times greater likelihood of being employed at baseline (95% CI = 1.02–7.85) and a 3.3 times greater likelihood of working overtime (95% CI =1.65–6.61). Occupation type did not modify the association between cancer and employment status (Model 3). Women who were 6–10 year cancer survivors had a 2.3 times higher likelihood of being employed at baseline (95% CI = 1.04–5.20) and overtime (95% CI = 1.44–3.85) than women without a history of cancer; however, if these women had professional jobs, they were less likely to work overtime than women with unskilled or manual jobs (OR = 0.40, 95% CI = 0.21–0.74) (Model 4). Health status indicators, such as fatigue and depression, were not associated with employment and had minimal impact on multivariable model estimates.

These changes do not affect the scientific results. We apologize for any inconvenience caused to readers or authors by this error. The article will be updated and the original will remain on the webpage.

## Figures and Tables

**Table 2:** Cancer and Employment Status among Women ^a^.

Variable	Model 1	Model 2	Model 3	Model 4
	Cancer	Years since Diagnosis	Cancer × Occupation	Years since Diagnosis × Occupation
	OR (S.E.) ^b^	OR (S.E.) ^b^	OR (S.E.) ^b^	OR (S.E.) ^b^
Fixed Effects				
Intercept (baseline level) ^c^	26.92 (4.58) ***	26.97 (4.59) ***	26.61 (4.54) ***	26.60 (4.54) ***
Cancer status (ref: no cancer)				
Has cancer	1.27 (0.19)		1.51 (0.34)	
Years since diagnosis (ref: no cancer)				
≤2 years		2.83 (1.47) *		1.43 (1.11)
3–5 years		1.03 (0.34)		1.49 (0.74)
6–10 years		1.61 (0.41)		2.33 (0.95) *
>10 years		0.94 (0.22)		1.13 (0.39)
Occupational status (ref: unskilled/manual)				
Professional occupations	2.57 (0.20) ***	2.56 (0.20) ***	2.61 (0.21) ***	2.62 (0.21) ***
Interaction Terms (cancer × occupation)				
Cancer × professional			0.79 (0.22)	
Interaction Terms (years since diagnosis × occupation)				
≤2 years × professional				2.55 (2.69)
3–5 years × professional				0.50 (0.30)
6–10 years × professional				0.68 (0.33)
>10 years × professional				0.78 (0.32)
Linear slope (ctime) ^d^	0.66 (0.02) ***	0.66 (0.02) ***	0.65 (0.02) ***	0.65 (0.02) ***
Cancer status (ref: no cancer)				
Has cancer	1.33 (0.12) **		1.59 (0.22) **	
Years since diagnosis (ref: no cancer)				
≤2 years		3.30 (1.16) **		1.91 (1.03)
3–5 years		1.07 (0.23)		0.87 (0.28)
6–10 years		1.34 (0.20)		2.35 (0.59) **
>10 years		1.18 (0.16)		1.45 (0.29)
Occupational status (ref: unskilled/manual)				
Professional occupations	0.77 (0.03) ***	0.77 (0.03) ***	0.79 (0.03) ***	0.79 (0.03) ***
Interaction Terms (cancer × occupation)				
Cancer × professional			0.74 (0.12)	
Interaction Terms (years since diagnosis × occupation)				
≤2 years × professional				2.20 (1.58)
3–5 years × professional				1.48 (0.58)
6–10 years × professional				0.40 (0.12) **
>10 years × professional				0.72 (0.18)
Random Effects				
Variance (ctime)	1.00 (0.08)	0.99 (0.08)	1.00 (0.08)	0.99 (0.08)
Variance (Intercept)	4.53 (0.26)	4.50 (0.26)	4.53 (0.26)	4.51 (0.26)
Covariance (ctime, intercept)	1.35 (0.11)	1.33 (0.11)	1.35 (0.11)	1.33 (0.11)
Log likelihood chi^2^(df)	779.53 (24) ***	789.37 (30) ***	782.62 (26) ***	798.20 (38) ***

^a^ Note: N = 7088 individuals; Observations = 26,355 observations (483 cancer survivors (1709 observations) and 6605 individuals with no cancer history (24,646 observations)). ^b^ Odds Ratios (Standard Error) are based on random slope logistic regression models adjusted for age, race/ethnicity, education, marital status, economic recession experience, children in the household, family income, type of health insurance, self-rated health, fatigue, depressive symptoms, and current cancer treatment; * *p* < 0.05. ** *p* < 0.01. *** *p* < 0.001; ^c^ Intercepts indicate person-specific baseline level (initial values) of employment status. ^d^ Linear slopes describe intra-individual patterns of change in employment status as a function of centered time (ctime).
