# Supplementary material for: Long-Term Employment Outcomes among Female Cancer Survivors
Source: Int J Environ Res Public Health. 2020 Apr 16;17(8):2751. doi: 10.3390/ijerph17082751 (PMC7215616; doi:10.3390/ijerph17082751)
Supplement: Supplementary file 1 [file ijerph-17-02751-s001.pdf]

Table S1. Effect of Length of Cancer Survivorship and Occupation Type on Odds of Employment among Cancer Survivors[a]

	Model 1 Years since diagnosis	Model 2 Years since diagnosis × occupation
**Variable**	OR (S.E.)[b]	OR (S.E.)[b]
**Years since cancer diagnosis (ref: ≤ 2 years)**		
3-5 years	.29(.14)*	.64(.51)
6-10 years	.37(.18)*	.69(.52)
>10 years	.24(.12)**	.52(.39)
**Occupational status (ref: unskilled/manual)**		
Professional types of occupation	1.19(.27)	3.69(3.37)
**Interaction Terms (years since diagnosis × occupation)**		
3-5 years × professional		.26(.27)
6-10 years × professional		.35(.34)
>10 years × professional		.29(.27)

[a]Note: N = 483 individuals; Observations = 1,709

[b]Odds Ratio (Standard Error) are based on random slope logistic regression models adjusted for age, race/ethnicity, education, marital status, economic recession experience, children in the household, family income, type of health insurance, self-rated health, fatigue, depressive symptoms, and current cancer treatment; * $p < .05$. ** $p < .01$. *** $p < .001$

Table S2. Effect of Length of Cancer Survivorship and Occupation Type on Weekly Hours Worked among Cancer Survivors[a]

	Model 1 Years since diagnosis	Model 2 Years since diagnosis × occupation
**Variable**	OR (S.E.)[b]	OR (S.E.)[b]
**Years since cancer diagnosis (ref: 1-2 years)**		
3-5 years	-5.96(2.65)*	-9.43(4.70)*
6-10 years	-5.58(2.49)*	-9.47(4.40)*
>10 years	-6.17(2.51)*	-5.18(4.32)
**Occupational status (ref: unskilled/manual)**		
Professional types of occupation	-3.02(1.38)*	-4.92(4.78)
**Interaction Terms (years since diagnosis × occupation)**		
3-5 years × professional		5.21(5.66)
6-10 years × professional		5.67(5.28)
>10 years × professional		-.93(5.09)

[a]Note: N = 333 individuals; Observations = 1,167

[b]Coefficients are based on random slope coefficient models adjusted for the covariates; * p < .05. ** p < .01. *** p < .001
